# Evaluation of novel compounds as anti-bacterial or anti-virulence agents

**DOI:** 10.3389/fcimb.2024.1370062

**Published:** 2024-03-06

**Authors:** Brankica Filipić, Dušan Ušjak, Martina Hrast Rambaher, Slavica Oljacic, Marina T. Milenković

**Affiliations:** ^1^ Department of Microbiology and Immunology, Faculty of Pharmacy, University of Belgrade, Belgrade, Serbia; ^2^ Laboratory for Molecular Biology, Institute of Molecular Genetics and Genetic Engineering, University of Belgrade, Belgrade, Serbia; ^3^ Department of Pharmaceutical Chemistry, Faculty of Pharmacy, University of Ljubljana, Ljubljana, Slovenia; ^4^ Department of Pharmaceutical Chemistry, Faculty of Pharmacy, University of Belgrade, Belgrade, Serbia

**Keywords:** novel compounds, antimicrobial resistance, anti-bacterial assays, anti-virulence assays, virulence factors, QSAR, molecular docking

## Abstract

Antimicrobial resistance is a global threat, leading to an alarming increase in the prevalence of bacterial infections that can no longer be treated with available antibiotics. The World Health Organization estimates that by 2050 up to 10 million deaths per year could be associated with antimicrobial resistance, which would equal the annual number of cancer deaths worldwide. To overcome this emerging crisis, novel anti-bacterial compounds are urgently needed. There are two possible approaches in the fight against bacterial infections: a) targeting structures within bacterial cells, similar to existing antibiotics; and/or b) targeting virulence factors rather than bacterial growth. Here, for the first time, we provide a comprehensive overview of the key steps in the evaluation of potential new anti-bacterial and/or anti-virulence compounds. The methods described in this review include: a) *in silico* methods for the evaluation of novel compounds; b) anti-bacterial assays (MIC, MBC, Time-kill); b) anti-virulence assays (anti-biofilm, anti-quorum sensing, anti-adhesion); and c) evaluation of safety aspects (cytotoxicity assay and Ames test). Overall, we provide a detailed description of the methods that are an essential tool for chemists, computational chemists, microbiologists, and toxicologists in the evaluation of potential novel antimicrobial compounds. These methods are cost-effective and have high predictive value. They are widely used in preclinical studies to identify new molecular candidates, for further investigation in animal and human trials.

## 1 Introduction

Antimicrobial resistance, caused by the misuse of anti-bacterial agents, is one of the top ten global public health problems associated with an alarming increase in the number of bacterial infections that can no longer be treated with available chemotherapies (Raffatellu, 2018). According to the WHO, at least 700,000 people worldwide currently die each year from antibiotic resistant infections. However, recent estimates suggest that in 2019, 4.95 million deaths were related to antimicrobial resistance and 1.27 million deaths were directly attributed to it (Murray et al., 2022). Given the current situation, the WHO estimates that by 2050, the number of human deaths due to bacterial infections could exceed 10 million per year, most of which are likely to be due to antimicrobial resistance (https://www.who.int/news-room/fact-sheets/detail/antimicrobial-resistance). To tackle this emerging health crisis, it is essential that novel antimicrobial agents are developed to spare existing arsenal of antibiotics. In addition, an alternative is to use approaches to disarm bacterial populations, without directly killing the infectious organisms, and/or sensitize them to the action of existing antibiotics.

One strategy to combat rising antimicrobial resistance is therefore to target the virulence factors of pathogenic bacteria. Virulence factors are specific molecules or structures synthesized by bacteria (Virulence Factor Database, http://www.mgc.ac.cn/VFs) that enable them to colonize, invade and persist in the host cell (Chen et al., 2005). If they are removed, this selectively impairs virulence without affecting viability. Virulence factors contribute to disease by either harming the host or evading the host’s immune system. Therefore, anti-virulence drugs have the capacity to decrease the need for antibiotics and lower the emergence and spread of antibiotic resistance. This is achieved while safeguarding beneficial, commensal microbiota without compromising the bacterial viability. These anti-virulence drugs are further classified into various groups, including adhesion and attachment inhibitors, toxin inhibitors, secretion inhibitors, communication and signaling inhibitors, among others (Ogawara, 2021; Gómez et al., 2022). Many virulence factors are involved in bacterial biofilm formation. Bacterial biofilms are clusters of bacteria that are surrounded by a matrix of extracellular polymeric substances (EPS) (Costerton et al., 1987; Flemming et al., 2016), that can colonize various surfaces. Most of the bacteria and fungi form biofilm following the adhesion to solid, living, or non-living surfaces, and less frequently in the liquid environment in the form of mobile aggregates (Vert et al., 2012). The EPS matrix is composed of polysaccharides, proteins, lipids, and extracellular DNA and provides a unique environment for various functions such as promoting intercellular bacterial communication and horizontal gene transfer, improving adhesion to surfaces, and allowing for digestion of nutrients. It also provides protection from external agents and prevents dehydration of bacteria (Jefferson, 2004; Flemming et al., 2007). The EPS matrix hinders the penetration of antimicrobials into the biofilm and accumulates cell products (e.g. catalase enzymes) which can then degrade the anti-bacterial drugs. Furthermore, the gradient of nutrients and bacterial metabolites in the biofilm results in areas where cells are in a dormant state, namely nongrowing cells with extremely reduced metabolic activity. These cells are highly resistant to traditional antibiotics that typically target growing and metabolically active bacteria (Lewis, 2001) and much higher doses (100 – 1000 fold) of antibiotics are required to eradicate bacteria in biofilm matrix (Costerton et al., 1999; Davies, 2003). The bacterial species *Staphylococcus epidermidis*, *Pseudomonas aeruginosa*, *Staphylococcus aureus*, *Klebsiella pneumoniae*, *Enterococcus faecalis*, *Streptococcus viridans*, *Escherichia coli* and *Proteus mirabilis* are frequently associated with diseases such as cystic fibrosis, ear and urinary tract infections, respiratory tract infections, diabetic ulcers, wounds and medical device associated infections that are exacerbated by biofilm formation (Del Pozo, 2018). In recent years, bacterial biofilms have been recognized as a serious threat to public health and safety (Flemming et al., 2007). In 2008, it was reported that 60% of chronic infections were caused by biofilms (James et al., 2008), moreover, the US National Institute of Health has reported that 80% of persistent infections in patients are associated with bacterial biofilms (Sharma et al., 2019).

Having all this in mind the development of novel antimicrobial agents that target virulence mechanisms rather than bacterial growth is recognized as promising strategy to reduce antimicrobial resistance. The use of *in silico* methods in combination with *in vitro* experiments is considered the most cost-effective way to discover new anti-bacterial and anti-virulence agents and to better understand their mechanisms of action.

In the recent decades, Computer-Aided Drug Design (CADD) methods have proven to be crucial for the identification of potential drug candidates for the treatment of various diseases. *In silico* drug design comprises two strategies: Ligand Based Drug Design (LBDD) and Structure Based Drug Design (SBDD). Both include different methods which are described in detail in the literature (Chang et al., 2023) and have been successfully used to design new compounds with antimicrobial and anti-virulence activity, and to understand key molecular interactions between drug and target (Yu and MacKerell, 2017). Here, we first discuss selected computational approaches and the principles of their work. Then, experimental methods for determining the anti-bacterial properties of new compounds are briefly discussed, followed by a comprehensive description of the methods used to determine the antibiofilm and/or anti-virulence properties of new anti-bacterial drug. In addition, some basic assays for assessing the safety and selectivity of new compounds are also described.

## 2 *In silico* methods for evaluation of novel compounds

### 2.1 Identifying novel anti-bacterial and anti-virulence agents using ligand based drug design approach

The Ligand Based Drug Design (LBDD) approach is based on the knowledge of active or inactive compounds that are already known to potentially interact with the target. This approach is very useful when the structure of the target protein is unknown. Quantitative Structure-Activity Relationship (QSAR) analysis is one of the LBDD methods commonly used to optimize the structure and properties of known compounds and to design new compounds with improved antimicrobial or anti-virulence activity. QSAR studies are based on the creation of mathematical models that establish a correlation between the chemical structure of the investigated compounds and their experimentally determined activity. To achieve this, the chemical structure of each compound must be described numerically in the form of a molecular descriptor. The first step in developing a QSAR model is to create relevant data set with the activity values of interest (Minimal Inhibitory Concentration - MIC, inhibition of biofilm formation) (Aleksić et al., 2017; Sathiyamoorthi et al., 2021; Boya et al., 2022) The compounds for QSAR analysis can be taken from various databases (e.g. ChEMBL - https://www.ebi.ac.uk/chembl/, PubChem - https://pubchem.ncbi.nlm.nih.gov), from published papers or synthesized and experimentally evaluated by the researchers themselves. The second step involves calculation of molecular descriptors, which in principle can be any molecular property. Numerous software (Dragon, CODESSSA, etc.) are available for the calculation of different types of molecular descriptors such as geometric, electronic, constitutional, topological, physicochemical descriptors (Guha and Willighagen, 2012). The next step is to select the key structural descriptors (independent variables, X) that influence biological activity (dependent variables, Y) by building QSAR models and using various statistical tools, such as Multiple Linear Regression (MLR), Partial Least Squares Analysis (PLS), Artificial Neural Network (ANN), Support Vector Machine (SVM). All created QSAR models need to meet strict validation criteria so that they can be reliably used to predict the antimicrobial or anti-virulence activity of newly designed compounds and to identify the most important structural features of examined compounds in establishing ligand-receptor interactions. Finally, the most active compounds, predicted by the model, need to be synthesized and their activity have to be confirmed by *in vitro* experiments (Bacilieri and Moro, 2006).

In the study by Verma et al. (2017), the authors reported the QSAR analysis of 17 synthesized 1,3-disubstituted-1*H*-naphtho[1,2-e] [1,3] oxazines, whose anti-bacterial activity against *S. aureus, Bacillus subtilis* and *E. coli* was evaluated. Three QSAR models were created, separately for each bacterium. Multiple linear regression was used to create the QSAR models, using the negative logarithm of the MIC values (pMIC) as the dependent variable and various calculated topological descriptors as independent variables. The predictive power of the developed models was confirmed by low residual values between the observed anti-bacterial activities and the activities predicted by the created models. Based on the QSAR models found, the authors identified important determinant for the anti-bacterial activities and then used them for the design of new oxazine derivatives. Decrease in value of third order molecular connectivity index descriptor leads to an increase in the anti-bacterial activity against *E. coli*. A decrease in the value of the Balaban index leads to an increase in the anti-bacterial activity against *S. aureus.* A decrease in the value of the topological descriptor (*kα_2_*) leads to an increase in the anti-bacterial activity against *B. subtilis.* Based on the selected descriptors, the authors designed new oxazine derivatives. For the designed compounds they calculated the molecular descriptors that appeared in the QSAR equations and used these equations to predict the pMIC values of the designed compounds. Obtained results showed that the designed compounds E (pMIC 2.92 against *E. coli*), F (pMIC 3.24 against *E. coli*) and H (pMIC 2.86 against *B. subtilis*) are more active than the synthetic compounds used to create the QSAR models. Designed compounds should be further synthesized and tested *in vitro*. Sathiyamoorthi et al. (2021) used a set of 16 halogenated indoles to determine the MIC values against *Vibrio parahaemolyticus.* The negative logarithm of the MIC (pMIC) was used as dependent variable in the creation of the 3D-QSAR model using the PLS method. The created QSAR model revealed that nucleophilic substitution, such as –Cl and –Br at position 4 and 5 of indole contribute to the enhancement of anti-bacterial activity against *V. parahaemolyticus.* The authors suggest that 4-chloroindole, 4-bromoindole, 5-chloroindole and 5-bromoindole (MIC 50 μg/mL) are the lead molecules for the development of novel anti-bacterial agents against *V. parahaemolyticus.* In addition, 4-chloroindole at a concentration of 20 mg/mL inhibited more than 80% of biofilm formation with a minimum inhibitory concentration (MIC) of 50 μg/mL against *V. parahaemolyticus* and *V. harveyi*. The set of indole derivatives was also the subject of the study by Boya et al. (2022). They carried out a 3D-QSAR analysis for 83 indole derivatives whose MICs were experimentally determined against uropathogenic *E. coli*. The MIC values of the investigated derivatives were converted into pMIC values [−log (MIC)] and used as dependent variable for the generation of 3D atom-based QSAR models using PHASE (Schrodinger software solutions). Five PLS factor models were created and the fifth model was selected for QSAR visualization and activity predictions. A 3D-QSAR analysis revealed that substitutions at the fourth and fifth positions of the indole moiety favored antimicrobial activity, while the seventh position had unfavorable effects. Similar to the previous study, the most promising indole derivatives were: 4-chloroindole, 5-chloroindole, and 5-chloro 2-methyl indole. They have a favorable *in silico* ADMET (Absorption, Distribution, Metabolism, Excretion, and Toxicity) profile and exhibited MICs of 75 μg/mL and inhibited biofilm formation by an average of 67% at 20 μg/mL. The experimentally determined values of percentage of inhibition of biofilm formation in *Serratia marcescens* were used by Aleksić et al. (2017) to create the QSAR model for 27 4-aminoquinoline derivatives. The calculated set of 180 molecular descriptors was used as the independent variable, while the logarithm of biofilm formation values measured at different concentrations (10, 25, and 50 μg/mL), denoted as log BI_10_, log BI_25_, and log BI_50_, respectively, were used as dependent variables. PLS regression was used for QSAR models building. The predictive power and optimal model complexity (number of PLS components) were estimated by double cross-validation. Two statistically significant models were obtained with log BI_25_ and log BI_50_ as dependent variables. In the case of log BI_10_, a narrow range of biofilm formation percentages (0.25 log units) was not sufficient to build a reliable QSAR model. Obtained QSAR models have shown that branching and size of molecules are the key topological descriptors responsible for modulating biofilm formation (Aleksić et al., 2017). These findings can be used for the design of new 4-aminoquinoline derivatives whose activity can be easily predicted using the established model, which may help in the identification of more effective compounds compared to reported 7-Cl and 7-CF_3_ substituted N-dodecylamino-4-aminoquinolines (inhibitors of biofilm formation with 50% biofilm inhibition at 69 μM in *S. marcescens*).

The paper of Cardoso et al. (2020) presents comprehensive review of the computational methods in designing antimicrobial peptides (AMPs) highlighting importance of the QSAR models in AMP sequence optimization and design of compounds with improved biological activities (Cardoso et al., 2020). One of the described QSAR models was successfully used to screen thousands of hypothetical peptide sequences *in silico*, leading to the development of novel antibiofilm peptides with enhanced activity against methicillin-resistant *S. aureus* (MRSA) biofilms which was confirmed by *in vitro* and *in vivo* experiments. Peptide 3002 (ILVRWIRWRIQW-NH2) exhibited an 8-fold higher antibiofilm potency *in vitro* than the parent peptide 1018 (Haney et al., 2018).

### 2.2 Identifying novel anti-bacterial and anti-virulence agents using structure based drug design approach

The Structure Based Drug Design (SBDD) approach relies on the 3D structural information of the target proteins and can be used in different stage of drug design. Among SBDD methods, molecular docking and structure-based virtual screening were successfully used to identified key sites and interactions important for understanding ligand biological function and to design antimicrobial or anti-virulence compounds that can compete with essential interactions involving the target and thus interrupt the biological pathways important for virulence potential or survival of the microorganisms. In order to perform molecular docking two essential requirements are needed: structural data for candidate ligands and 3D structure of target proteins which along with an adequate molecular docking algorithms available to predict protein-ligand poses and to rank them based on scoring function enable selection of the most promising ligands for further optimization and synthesis (Elokely and Doerksen, 2013). Experimental methods, such as X-ray crystallography, nuclear magnetic resonance spectroscopy and cryo-electron microscopy have provided valuable 3D information on many target protein and ligand-target complexes which are accumulated in Protein Data Bank (PDB) repository (https://www.rcsb.org/) representing the main source of experimentally-determined 3D structures as well as computed structure models of target proteins. When experimentally determined 3D structure is not available, homology modelling which depend on finding an experimentally determined protein structure with similar sequence to use as a modelling template can be applied, or protein structure can be predicted using artificial intelligence and machine learning approaches when template is not available (Chang et al., 2023). The candidate ligands are usually small molecules whose number rapidly increase in available synthesized chemical libraries. The *in silico* databases of drug-like compounds, such as ZINC database (https://zinc.docking.org) are essential for ligand identification based on virtual screening process.

In recent years, molecular docking as a part of virtual screening of large databases have been used for selecting potent antibiofilm agents targeting some of regulatory proteins involved in biofilm formation, such as diguanylate cyclase PleD (Fernicola et al., 2016), biofilm-associated protein (Bap) produced by *Acinetobacter baumannii* (Tiwari et al., 2018), the ribosomally-associated enzyme RelA (Hall et al., 2020), LasR, a transcription factor that controls QS in *P. aeruginosa* (Kalia et al., 2017), the pqs QS receptor PqsR (also known as MvfR) (Mellini et al., 2019). In the study by Fernicola and coworkers (Fernicola et al., 2016), two novel molecules with a catechol moiety and a sulfonohydrazide scaffold were identified as potent inhibitors of the diguanylate cyclase PleD from *Caulobacter crescentus*, which are considered attractive molecular targets for the development of antibiofilm agents. The 3D structure of the target protein PleD was taken from the PDB (PDB ID: 2V0N). Based on the binding mode of the substrate analog GTP-α-S (guanosine-alpha-thio-triphosphate) bound to PleD, the key chemical features responsible for the main binding interactions were derived using a pharmacophore-based approach. The resulting pharmacophore was used to screen the ZINC database, and the most promising virtual screening hits were selected based on their predicted affinity for PleD and their commercial availability. Two of the 13 compounds tested *in vitro* with the highest predicted affinity (compounds 2 and 7) inhibit PleD in the low-micromolar range (50% inhibitory concentration [IC_50_] of ~11 µM) and could be used as lead compounds for the development of new anti-virulence drugs that act on c-di-GMP signaling. Tiwari et al. (2018) used the biofilm-associated protein (Bap) produced by *A. baumannii* as a target protein for the discovery of effective antibiofilm molecules. Homology modelling was performed to generate the 3D structure of the protein Bap (396aa) since no structure is available in the protein database. Amino acid residues within 4 Å of the active site were used to generate the receptor grid of Bap, which was used for virtual screening of 2924 Food and drug Administration (FDA) approved drugs from the ZINC database and Ligprep. After ADMET and Lipinski filtering, 228 compounds were selected and subjected to docking analysis. In this *in silico* study, ZINC00039089 (L-Adrenaline) was identified as an inhibitor of Bap of *A. baumannii*. The selected drug was further experimentally validated. The IC_50_ was calculated based on the 50% reduction in biofilm formed by strain RS-307 of *A. baumannii*. The result showed that adrenaline exhibited antibiofilm activity with an IC_50_ value of 75 µg/ml (Tiwari et al., 2018). A virtual screening approach was also used to identify FDA-approved drugs targeting the pqs Quorum sensing (QS) system of *P. aeruginosa* using *in silico* molecular docking (Mellini et al., 2019). The simulations were performed with the apo form of PqsR (PDB ID: 4JVC). Of the five selected hits, the antipsychotic pimozide provided the most promising experimental results in terms of pyocyanin production, swarming motility and biofilm formation. A recent study by Dighe et al. (2021) presented the discovery of novel antimicrobial agents using a virtual screening approach based on a combination of docking and pharmacophore methods. They searched the CoCoCo database, which contains 1.4 million compounds, for new ClpP inhibitors (proteolytic subunit of caseinolytic protease). ClpP is a serine protease that maintains bacterial homeostasis through the controlled degradation of short-lived regulatory proteins as well as misfolded or damaged proteins. The docking-based virtual screening was performed with ClpP from the protein data bank (PDB ID: 5DL1). The grids for the docking simulations were generated using the structural coordinates of the bound co-crystallized ligand 4 [1-(1-isopropyl-1H-indazol-5-yl)-N-((2-(thiophen-2-yl) oxazol-4-yl) methyl) methan- amine]. After applying a Virtual Screening Workflow (VSW) protocol consisting of three docking protocol steps, i. e., high throughput virtual screening (HTVS), standard precision (SP), and extra precision (XP) and two pharmacophore screening protocol steps (firstly against the pharmacophore developed from the receptor-ligand complex (ClpP X-ray crystal structure, PDB: 5DL1) and secondly against the pharmacophore developed from a non-covalent ClpP inhibitor 4) based on binding orientation and interaction within the active site of ClpP, a final group of 12 molecules was selected to evaluate their bactericidal activity *in vitro.* Compound 6 (benzimidazole derivative) proved to be the most potent with MIC values between 4–16 μg/mL against methicillin-sensitive *S. aureus* (MSSA) and MRSA strains. Based on the docking studies performed, the authors hypothesized that compound 6 could be a non-covalent inhibitor of ClpP and served as a lead structure for the development of a new class of antimicrobial agents. Based on compound 6, the authors then performed a 75% substructure similarity search on SciFinder and obtained 15 commercially available analogues with variations on the benzimidazole ring, aliphatic linker, thiazole ring, and diflurophenyl ring of compound 6. After their *in vitro* evaluation compound 24 found to be the most potent analogue of the series (MIC 4 µg/mL against three *S. aureus* strains) and found two times more potent than standard antibiotics, gentamicin, and trimethoprim, against two MRSA strains (MRSA 1113 and MRSA ATCC 33591). In addition, compound 24 has satisfactory *in silico* pharmacokinetic properties and no cytotoxicity in two human cell lines. Further studies should be conducted to determine ClpP inhibitory potential and to validate the drug target (Dighe et al., 2021).

Molecular docking studies were also carried out to verify and rationalize anti-bacterial properties of some synthesized compounds with promising MIC and MBC values indicating on possible structural modification for achieving better anti-bacterial activity. Some examples include molecular docking studies of synthesized pterostilbene derivatives against DNA polymerase (Tang et al., 2019), fluoroquinolones derivatives against *E. coli* DNA gyrase B (Fekadu et al., 2022), quinoline derivatives against *S. aureus* tyrosyl-tRNA synthetase (Bouzian et al., 2020), diclofenac derivatives against DNA gyrase (Hamed et al., 2023).

Despite numerous papers exploring potential anti-virulence agents, effective commercial drugs that are widely used in clinical practice are still lacking. Only a few immunoglobulin drugs (BabyBIG, BAT, Raxibacumab, Obiltoxaximab, Bezlotoxumab) have been approved by the FDA as anti-virulence drugs (Dickey et al., 2017). Predicting the development of bacterial resistance is also difficult, as bacterial transmission and colonization are complex and incompletely understood (Dickey et al., 2017).

Undoubtedly, CADD methods are time and cost efficient in identifying lead compounds with potential anti-bacterial and anti-virulence activity. They are also very important for better understanding of their molecular mechanism of action. Despite the validation protocols that are an integral part of CADD, many computationally discovered compounds have not confirmed their efficacy in *in vitro* and *in vivo* experiments. Nevertheless, CADD is an important component in the discovery of new compounds with anti-bacterial and anti-virulence activity.

## 3 Evaluating anti-bacterial activity of novel compounds

### 3.1 Determination of bacteriostatic or bactericidal effect of novel compounds

The Minimal Inhibitory Concentration (MIC) assay is used to determine the lowest concentration of an antimicrobial agent required to inhibit visible *in vitro* growth of a specific microorganism and is usually the first step in evaluating the antimicrobial potential of novel compounds (Balouiri et al., 2016). There are several methods for MIC determination which have been described in detail elsewhere and are also described in the guidelines of the European Committee on Antimicrobial Susceptibility Testing and the Clinical and Laboratory Standard Institute (CLSI) guidelines (Andrews, 2001). The most commonly used methods for evaluating the antimicrobial properties of new compounds are the broth dilution and agar dilution method (Wiegand et al., 2008). In the broth dilution method, the MIC is determined by subjecting the antimicrobial agent to a half-dilution in conjunction with the specific bacteria, typically at a suspension density of 10^5^ colony forming units (CFU) per millilitre (mL). The assessment of microorganism growth relies on visually inspection of the turbidity or by measuring the optical density (OD) at 600 nm and is expressed in mg/L (or µg/mL) (Reller et al., 2009). The MIC assay is imperative to conduct the Minimal Bactericidal Concentration (MBC) for a new agent. The MBC is assessed by subculturing the broths used for MIC determination onto fresh agar plates. The MBC represents the lowest drug concentration that leads to the demise of 99.9% (3 logarithms) of the bacteria and enables determination of the minimum concentration of new anti-bacterial agent necessary to achieve bactericidal effect. If the MBC does not exceed four times the MIC, the anti-bacterial agent is classified as bactericidal.

A more advanced assay for evaluating bacteriostatic or bactericidal effect is the time-kill kinetic assay which is described in detail in the CLSI M26-A document (https://clsi.org/standards/products/microbiology/documents/m26/). This assay allows the analysis of the impact of varying concentrations of an anti-bacterial agent over time, in relation to the different stages of the specific bacteria’s growth (lag, exponential, and stationary phases). Briefly, the assay is performed by adding a new anti-bacterial agent to media containing bacteria, then determining the logarithmic colony forming units per millilitre (CFU/mL) at different time intervals using the plate counting method. Time-kill assays are often used to ascertain synergism between two or more antibiotics, and to determine whether an antibiotic exhibits a time- or concentration-dependent effect. In time-dependent killing, the bactericidal effect persists as long as the concentration of the anti-bacterial agents remains above the MIC (Ferro et al., 2015). A concentration-dependent effect occurs when the bactericidal activity increases with the higher concentration of the antibiotic.

### 3.2 Determination of post antibiotic effect

The post antibiotic effect (PAE) is defined as a period following the complete elimination of an antibiotic, during which the target organism does not experience any growth. This phenomenon is observed in a wide range of antimicrobial agents and has been well-documented in various prevalent bacterial pathogens. Several variables, such as the microbial strain, the specific antimicrobial agent, its concentration, the duration of exposure, and the combination with other antimicrobials, play a role in determining the presence and duration of the PAE (MacKenzie and Gould, 1993). Several methods for the determination of PAE have been described. Most researchers determine the PAE *in vitro* by exposing the broth culture to an antibiotic at concentrations above the MIC for at least one hour. Subsequently, the drug is removed using various techniques, usually by centrifugation. After removal of anti-bacterial drug, serial samples are collected, and viable count is performed. This method was established by McDonald and colleagues in 1977 and the simple formula has been set up as PAE = T – C, where T is the time required for the treated bacteria to achieve 10-fold growth after washing out the antibiotic, and C is the time needed for the untreated bacteria to increase 10-fold after washing with fresh medium. This method can be applied to all antimicrobials (Zhanel et al., 1991), but it is labor intensive, therefore other methods have been developed. The most convenient are the spectrophotometric methods, in which the bacteria are resuspended in fresh medium and added to the microtiter plates, followed by automatic measurement of the turbidity of the culture every 10 minutes. The PAE was calculated as the time taken for antimicrobial drug treated cultures to reach 50% of the ODmax of the control culture, minus the time required for the control culture to reach the same point (Stubbings et al., 2004).

### 3.3 Development of resistance to novel compounds

The potential of bacteria to evolve resistance to a new agent is important information for public health and especially in development of new potential anti-bacterial drug. Most assays to assess bacteria’s tendency to develop resistance to an antibiotic through spontaneous mutation based on a serial passage experiment (Martínez et al., 2011). In this assay, multiple populations are routinely transferred to media containing increasing concentrations of the new anti-bacterials. Mutations that enhance resistance generally lead to improved fitness at lower antibiotic concentrations. Thus, when bacteria are exposed to relatively low doses of antibiotics, the prevalence of mutations that confer resistance at higher concentrations is effectively increased (Bell and MacLean, 2018). In addition, mutations can be identified by fluctuation testing. In this approach, numerous separate cultures are seeded with a mutant-free inoculum of a parent strain. These cultures are then screened for resistance by plating on agar plates containing a high concentration of an antibiotic. The frequency of mutations that lead to high resistance can be approximated by counting the number of resistant colonies that emerge after a short incubation period of usually 1 to 2 days. In addition, sequencing techniques can be used to determine these mutations precisely.

In recent years, there has been a growing interest in researching and developing new antimicrobial agents. In 2023, according to the PubMed database, more than 5000 papers were published describing new anti-bacterial agents. Kokot and coworkers (2023) reported the development of novel bacterial topoisomerase inhibitors, and for all new compounds, the MIC was determined, followed by an in-depth microbiological evaluation of the most promising compound. The oxadiazole antibiotics have been published as bactericidal agents against *Clostridioides difficile*, and their anti-bacterial properties were evaluated by time-kill assay, post-antibiotic effect, and emergence of resistance (Qian et al., 2023). Cannabidiols have been very recently published as broad-spectrum anti-bacterial agents, and their lead compound acted as a bactericidal agent through a membrane-targeting mechanism with a low resistance frequency (Fang et al., 2024). Benzopyridone cyanoacetates have been reported as a new type of broad-spectrum anti-bacterial with low MIC values against several tested strains, bactericidal mode of action, and a low resistant trend (Zhang et al., 2024). The anti-bacterial compound MA220607 was published as dual-targeting inhibitor, facilitating FtsZ polymerization and perturbing bacterial membranes. It has a broad-anti-bacterial spectrum with a low incidence of drug resistance, low hemolytic activity, and good anti-bacterial efficacy *in vivo* (Ma et al., 2024).

## 4 Anti-virulence potential of novel compounds

### 4.1 Impact of novel compound on biofilm formation

Biofilm, both structurally and functionally, provides stability and persistence to the embedded microbial cells (Hall and Mah, 2017; Koo et al., 2017). Structural integrity, viscoelasticity, sustainability, and heterogeneity provided by the specific composition of the biofilm matrix contribute to the physical resilience of the biofilm (Stewart and Franklin, 2008; Flemming and Wingender, 2010; Peterson et al., 2015; Flemming et al., 2016). Besides, the biofilm is equipped with several additional mechanisms of resistance and tolerance, such as limited diffusion of antimicrobial agents, reduced metabolism in a portion of the biofilm cells, hypoxia, and highly increased horizontal gene transfer rate (Borriello et al., 2004; Lewis, 2010; Madsen et al., 2012; Tseng et al., 2013). Altogether, owing to these attributes, the biofilm facilitates the colonization of hospital surfaces and medical devices, as well as host tissues, and contributes to the difficult eradication of pathogens. This way, biofilm promotes hospital outbreaks, as well as pathogenicity and virulence of nosocomial pathogens (Colquhoun and Rather, 2020).

In addition to the hospital environment, all water systems in the pharmaceutical industry are susceptible to biofilm formation if not properly controlled. This can be caused by low-quality materials used for pipework and inappropriate diameter pipes or poorly designed bends, both of which can slow the rate of circulating water (Gilmore, 2023). To assess the effectiveness of a water system, microbiological testing of the water is required. A range of rapid methods is available for the screening of water samples for indicators of contamination based on chromogenic, fluorogenic, or chemiluminogenic substrates. An alternative approach is with light scattering methods which can be used for the detection of water pathogens (Gilmore, 2023).

**Figure 1:**
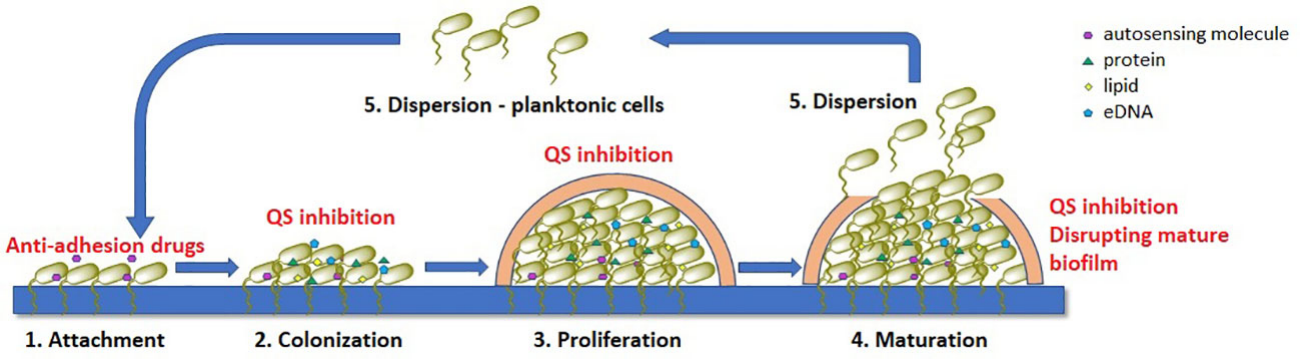
Stages of bacterial biofilm formation and the most common mechanisms of antibiofilm acting compounds (marked with red), QS – quorum-sensing.

Biofilm formation involves five interconnected stages, as outlined in **Figure 1** (Flemming et al., 2016; Srinivasan et al., 2021; Nadar et al., 2022).

1. Attachment: This is the initial stage in biofilm formation, where free-swimming planktonic cells attach to a surface through weak interactions such as acid-base, hydrophobic, Van der Waals, and electrostatic forces. Cell structures such as pili, flagella, or fimbriae also allow for mechanical adhesion to the surface. This stage is crucial as it allows the bacteria to establish a foothold on the surface, and it is also reversible, meaning that the cells can detach from the surface if conditions are not favorable for growth.
2. Irreversible colonization: Once the cells have attached to the surface, they begin to colonize it by producing extracellular matrix components such as collagen-binding proteins, lipopolysaccharides, flagella, and pili. These components help to anchor the cells to the surface and create a more stable environment for growth. This stage is irreversible, meaning that the cells cannot detach from the surface once they have colonized it.
3. Proliferation With the surface now colonized, the cells begin to multiply and form multi-layered clusters. This is accompanied by the production of EPS matrix, a complex mixture of polysaccharides, proteins, lipids, and extracellular DNA (eDNA), which surrounds the cells and forms a protective barrier. This matrix also helps to support the structural integrity of the biofilm.
4. Maturation: The multi-layered clusters of cells continue to grow and mature, forming a three-dimensional structure with distinct layers of cells. The extracellular matrix thickens and becomes more complex, further protecting the cells from external threats.
5. Dispersion: In the final stage, colonies of cells release individual planktonic cells that can travel to a new surface and start the cycle again. This allows the bacteria to spread to new locations and potentially colonize new surfaces.

All these stages play a crucial role in the formation of bacterial biofilms. Understanding these processes can help in the development of new strategies to combat biofilm-associated infections. We now know that in the treatment of chronic bacterial infections existing antibiotics have been selected for their efficacy against planktonic organisms and are often much less effective against biofilms.

There are several strategies that can be used to inhibit biofilm formation including: a) preventing bacteria from adhering to surfaces, b) disrupting bacterial communication through the use of quorum-sensing inhibitors (QSI), c) altering the concentration of signaling molecules, involved in biofilm formation and (d) inhibiting or disrupting mature biofilms (Ghosh et al., 2020; Asma et al., 2022). These approaches have shown promising results in terms of reducing the presence of pathogens, particularly when used in combination with traditional antibiotics. By disrupting the EPS matrix and preventing the formation of biofilms, the effectiveness of conventional antibiotics can be improved, and the spread of antibiotic resistance can be reduced. Furthermore, alternative treatments that target the virulence of bacteria within biofilms without directly killing the organisms can also be explored. Addressing bacterial biofilms is a crucial step in the fight against antimicrobial resistance and the development of novel biofilm inhibition methods should be a priority in research.

#### 4.1.1 Antibiofilm activity of novel compounds

Examination of the antibiofilm activity of novel compound can comprise evaluation of its influence on the degree of biofilm production, as well as on the biofilm structure and related functions. The most widely used is a microtiter plate colorimetric assay based on staining the biofilm with crystal violet (or safranin) (Stepanović et al., 2000, 2007). This method enables simple and cost-effective quantification of biofilm production and is suitable for high-throughput optimization. Briefly, after incubation of bacteria in the presence of a suitable growth medium supplemented with a tested compound, the wells are washed to remove planktonic cells, and the remaining biomass is stained with crystal violet. The biomass that is quantified comprises both live and dead biofilm cells, as well as some components of the biofilm matrix. However, some of the planktonic cells adhering to the surface or sedimented at the bottom due to the influence of gravity may also withstand the washing process and be included in the final result. On the other hand, a significant fraction of biofilm biomass may be lost during the washing procedure, especially for microbial species that form loose biofilm structures under the relevant experimental conditions, adding to the variability between independent replicates, so this method still cannot be standardized (Azeredo et al., 2017). To minimize the level of detachment during staining and rinsing of unbound dye, fixation process is applied by overnight incubation of a washed biomass with methanol, or by incubation at 60°C for 1 h. Another disadvantage is that biofilm production is only assessed on a polystyrene surface of a standard microtiter plate, although it is known that biofilm levels and structures largely depend on a surface type. This can be partially bypassed by coating wells or by incubating biofilm producing microorganisms in the presence of target surfaces (Filipović et al., 2021). Another option is to use Calgary biofilm device which is based on the use of coverlids equipped with pegs, commercially available in different material coatings (e.g. cellulose, titanium dioxide, hydroxyapatite, etc.), on which the biofilm is formed upside-down and therefore the influence of gravity and planktonic cells sedimentation is minimized (Ceri et al., 1999). The described methods can also be used for the quantification of polymicrobial biofilm production (Ušjak et al., 2023), as well as for the evaluation of biofilm eradication activity of novel compounds, when the compounds are added and incubated with the biofilm at different time intervals, after the biofilm has been fully formed and washed from the planktonic cells (Sabino et al., 2022).

The viability of treated biofilm cells can also be estimated by serial plating and CFU counting of washed and physically detached biofilm cells by vigorous vortexing or ultrasound treatment. This method additionally allows the quantification of individual members in polymicrobial communities when used in conjunction with selective agar media seeding (Filipović et al., 2021). However, it does not include viable but non-culturable cells (VBNC) and it may be impossible to detach and quantify the entire biomass, especially in the case of strongly adhesive biofilm structures (Li et al., 2014). Alternatively, biofilms incubated and washed according to standard microtiter plate procedure can be stained with tetrazolium salts, or resazurin, instead of crystal violet, to assess biofilm cell viability, including VBNC (Sabaeifard et al., 2014; Van den Driessche et al., 2014; Ramage, 2016). Other possible techniques include flow cytometry of differentially labelled total, dead and VBNC cells, as well as propidium monoazide linked quantitative PCR (PMA-qPCR) which allows selective quantification of DNA derived from live cells (Oliveira et al., 2015; Tavernier and Coenye, 2015). Both methods are also suitable for optimization to quantify individual members in polymicrobial biofilms. However, PMA-qPCR is not compatible for the evaluation of antibiofilm activity with novel compounds that affect the integrity of cell membranes and thus allow PMA to intercalate into the DNA of live cells as well (Sträuber and Müller, 2010).

Biofilm production levels, as well as the spatial structures and associated functions can be assessed using various microscopy techniques. The simplest and most cost-effective is quantification using light microscopy of *ex vivo* stained (e.g. hematoxylin and eosin (H&E), Brown and Brenn Gram, or periodic acid-Schiff (PAS) staining) infected tissue samples (Davis et al., 2008; Bulut et al., 2014). Analysis of biofilm spatial structure as well as biofilm volume, thickness and roughness can be performed using confocal laser scanning microscopy (CLSM) on labelled biofilm cells, which can also be optimized for distinction of individual members in mixed-species communities (Bridier et al., 2010; Neu and Lawrence, 2014). Also, various modifications of scanning electron microscopy (SEM) and atomic force microscopy have also been developed to enable the imaging and visualization of biofilm structures at higher resolution (Azeredo et al., 2017).

Finally, above listed *in vitro* static biofilm production assays exhibit limitations regarding the amount of biomass production and do not account for nutritional depletion and hydrodynamic conditions, which can significantly affect biofilm production in real-life environments. This may be bypassed with the use of dynamic biofilm production systems such as the modified Robbins device, drip flow biofilm reactor and various rotating biofilm reactors (Linton et al., 1999; Goeres et al., 2009; Coenye and Nelis, 2010; Pavarina et al., 2011). These systems rely on the use of disposable coupons which are commercially available made of various materials and they are not suitable for high-throughput analyses. The major disadvantage of these systems is that they do not allow real-time monitoring of biofilm development, which can be bypassed with the use of open or closed type devices, specifically designed to be compatible with different microscopy techniques (Azeredo et al., 2017).

Various biofilm production assessment techniques have been successfully implemented to identify novel compounds with significant antibiofilm activity. For example, Garcia et al. (2021) tested the most active chalcone derivative (out of a total of 17 synthesized chalcone compounds) against biofilm production of MSSA and MRSA. They tested the effects on both initial biofilm formation and preformed biofilms, and they also used quantitative assays to measure CFU to determine the number of viable cells in the treated samples and SEM to get high-resolution, three-dimensional images of the treated biofilm structures. Using these techniques helped them discover that the tested compound significantly reduced both biofilm formation and survival, surpassing the efficacy of vancomycin, which served as a control. Further, SEM confirmed the destructive effects on MSSA and MRSA biofilms, disrupting the architecture and reducing biofilm populations. In another study, the authors synthesized series of 31 different aniline derivatives and tested them against biofilm production of *E. faecalis* and *S. aureus* using biofilm eradication studies and Calgary biofilm device experiment to determine the minimum biofilm eradication concentration (MBEC). They found that the tested compounds exhibited good biofilm-killing abilities against both tested bacteria, with MBEC values ranging from 6.25 to 25 μg/mL, and that one compound showed better activity against *S. aureus* compared to vancomycin (Saleh et al., 2021). Further, Nithya et al. (2011) tested antibiofilm activity of novel compounds derived from the marine bacteria *Bacillus indicus* and *Bacillus pumilus* against 10 Gram-positive and Gram-negative bacterial species. Following the determination of biofilm inhibitory concentrations, they used light microscopy to observe treated biofilms, as well as CLSM to investigate biofilm damaging pattern of the most active compound. They demonstrated that tested compounds reduced cell surface hydrophobicity and that the selected compound significantly reduced the thickness of several biofilms. In addition, the use of dynamic biofilm formation procedure is exemplified in a paper investigating antibiofilm effectiveness of the limonene. Here, the authors used the CDC biofilm reactor, which is a type of rotary biofilm device, to simulate *in vivo* biofilms and investigated the efficacy of limonene in inhibiting biofilm formation by *S. aureus*, *P. aeruginosa*, and their mixed biofilm. They demonstrated that limonene exhibited significant inhibition and eradication of biofilms on polypropylene, polycarbonate, and steel surfaces, by measuring CFU/cm^2^ on each of these materials. They further showed that limonene modulated the expression of key genes (*eno*, *icaA*, *pilA*, *flgK*), revealing its impact on biofilm-related pathways in both microorganisms, highlighting its potential as a biofilm-disrupting agent (Gambino et al., 2022).

The development of antibiofilm agents faces challenges due to the complex and dynamic nature of biofilms. One key challenge is the heterogeneity of biofilm structures, which makes uniform penetration and action of antibiofilm agents difficult (Stewart and Franklin, 2008). In addition, the presence of EPS within biofilms can act as a protective barrier, limiting the accessibility of compounds to bacterial cells (Flemming and Wingender, 2010). The effectiveness of anti-virulence drugs in the clinical setting may be influenced by diverse microbial communities and the potential for microbial adaptation, posing a risk of resistance development and reduced long-term efficacy (Orazi and O'Toole, 2019). Addressing these challenges requires a comprehensive understanding of biofilm biology and the development of strategies that take into account the intricate interactions within biofilm communities.

### 4.2 Anti-quorum sensing potential of novel compounds

Quorum sensing (QS) was first discovered and described in the 1970s by Nealson et al. (1970) in two luminous marine bacterial species *Vibrio fischeri* and *Vibrio harveyi* and has been found in many Gram-negative and Gram-positive bacteria (Nealson et al., 1970; Abisado et al., 2018).

QS is a bacterial cell-cell communication system that enables bacteria density-dependent processes such as biofilm formation, development of genetic competence, antibiotic production, production of virulence factors (proteases, toxins, and adhesins), sporulation, conjugation, production of secondary metabolites and stress adaptation (Ogawara, 2021; Wu and Luo, 2021). QS is mediated by low molecular weight extracellular signaling molecules called autoinducers (AIs) that regulate bacterial gene expression. This chemical communication between bacteria is essential for survival, bacterial growth, and nutrient uptake (Dehbanipour and Ghalavand, 2022). Both Gram-positive and Gram-negative bacteria utilize QS for bacterial communication, although the types of QS pathways differ. Gram-positive bacteria use peptides, called autoinducing peptides (AIPs) as signaling molecules. When the extracellular concentration of AIPs is high, they activate membrane-bound two-component sensor histidine kinases. Transcription factors in the cytoplasm are phosphorylated by the kinase and then regulate gene expression (Monnet and Gardan, 2015; Wu and Luo, 2021; Dehbanipour and Ghalavand, 2022). In Gram-negative bacteria QS system has several common features. First, AIs are synthesized from S-adenosylmethionine (SAM) as a substrate and the most common class of autoinducers are acyl-homoserine lactones (AHLs). Second, AIs can freely diffuse across the bacterial membrane and to bind to specific receptors in the inner membrane or cytoplasm. Third, QS regulates dozens to hundreds of genes that underpin biofilm formation, virulence factor production and other various biological processes. Fourth, in a process called autoinduction, the QS molecule receptors establish a feed-forward loop that promotes synchronous gene expression in the bacterial population (Papenfort and Bassler, 2016; Wu and Luo, 2021). In other words, as the bacterial population grows, the level of AIs increases proportionally, and after reaching a certain level, the AIs diffuse back into the bacteria and regulate the transcription of genes responsible for biofilm formation, the expression of virulence factors and the production of antibiotics (Asfour, 2018). In addition to AIP and AHLs, there is a third class of signaling molecules called AI-2 which are used by both Gram-positive and Gram-negative bacteria for interspecies communication (Asfour, 2018).

QS has become an attractive target for the development of novel anti-microbial agents against resistant bacteria as anti-QS compounds can inhibit bacterial pathogenicity. Interfering with QS represents a so-called anti-virulence strategy for the treatment of bacterial infections. The development of QSIs is a way to expand the arsenal of anti-infective agents in addition to conventional antibiotics and antimicrobial agents. The mechanisms of QSIs can be diverse including inhibition of AIs, blocking the AIs receptor by antagonism, degradation of AIs, inhibition of AIs transport etc (Malešević et al., 2019; Dehbanipour and Ghalavand, 2022). The simplest approach for screening QSIs is the biological assay based on natural strain *Chromobacterium violaceum*, Gram-negative bacterium that can produce purple pigment violacein and therefore form purple colonies on common laboratory media (Lu et al., 2022). The violacein pigment is encoded by the *vio* operon and the expression of this pigment is QS-regulated, which is why these bacteria are often used as a model system for screening of QSIs in the laboratory (Kothari et al., 2017; Dimitrova et al., 2023). The loss of the pigment, in turn, indicates inhibition of QS, and those compounds that induce inhibition of the violacein production, without affecting bacterial growth, can be considered as true QSIs. The most common *C. violaceum* standard strains used in the screening assays are *C. violaceum* ATCC 12472 and *C. violaceum* ATCC 31532. Another *C. violaceum* strain frequently used in QS studies is *C. violaceum* CV026. CV026 is violacein-negative mini-Tn5 mutant of *C. violaceum* ATCC 31532 that lacks *cviI* encoded AHL synthase and thus violacein production can be restored in the presence of externally supplied AHLs. Compounds active only against CV026 (and not against standard *C. violaceum* strains) interfere with degradation of AHLs and are classified as quorum quenchers (QQs) (Manner and Fallarero, 2018; Lu et al., 2022).

According to the literature data various chemical compounds were evaluated for anti-QS potential and revealed positive effects. In the study of Ragab et al. (2023) 16 pyrazole and pyrazolo[1,5-a] pyrimidine derivatives were synthesized and assessed for their anti-QS activity. Five compounds named 3a, 5a, 6, 9a, and 10a were selected as they exhibited significant anti-bacterial activity. These five derivatives were evaluated for the anti-QS activity and the presence of these compounds reduced the violacein production by CV026, confirming their anti-QS activity (Ragab et al., 2023). Besides anti-QS activity tested compounds exhibited potent antibiofilm and anti-bacterial activity. In another study, novel synthetic compounds (18) from 2 series (Pyrazole and Diene dione) were screened for QS and biofilm inhibitory potential against resistant pathogens isolated from patients with chronic sinusitis (Rafiq et al., 2022). Findings within this study revealed that most novel compounds were effective anti-bacterial agents, some compounds were potent inhibitors of biofilm formation, whereas compound UA3 has shared significant anti-quorum sensing potential against *Chromobacterium pseudoviolaceum* (Rafiq et al., 2022). In the study performed by Bernabè et al. (2022) in house library of phenolic derivatives (81 compounds) were tested for anti-QS activity. The library contained monocyclic phenol derivatives and fused polycyclic phenols (cumarines and chromones). The experiments revealed that compound designated as GM-50 was the most active and inhibited the expression of AHL-regulated genes. GM-50 also reduced virulence factors such as rhamnolipids, pyocyanin, elastase secretion, and swarming motility in *P. aeruginosa* PAO1 laboratory strain (Bernabè et al., 2022).

In addition to the most used biological assay with *C. violaceum*, various approaches and strategies for QSI screening have been developed in recent years such as a) *in vivo Caenorhabditis elegans* models; b) chemical approaches to interrogating QS pathways for QSI discovery; c) antibodies for quenching QS signaling; d) virtual screening for the detection of QSIs; e) three-dimensional (3D) printing etc (Lu et al., 2022).

There is no doubt that the concept of anti-QS strategy (also called quorum quenching (QQ)) has a promising application in the fight against various types of pathogens, indicating the possibility that QQ reduces pathogenicity of the tested microorganisms and facilitates their eradication. On the other hand, anti-QS therapies have some challenges and limitations. The first objection is the selectivity of QQ substances. It should come as no surprise that QQ substances can have an indirect/direct effect on the ability of the human microbiota to adhere, form biofilms, and produce metabolites with antimicrobial activity, thus leading to a disruption of microbiota homeostasis (Krzyżek, 2019). The second objection relates to the reduction in the virulence of pathogens. There is evidence that the disruption of genes responsible for QS activity leads to an increase in certain pathogenicity traits. For example, deletion of *luxS* (*ΔluxS*) in certain microorganisms (e.g. *Helicobacter pylori, Vibrio cholerae, S. aureus, E. faecalis*) has increased aggregation or/and biofilm formation (Krzyżek, 2019). The third objection is the possibility of developing resistance to QQ therapies. The first study, using computer modeling, indicated the possibility of developing resistance to QQ molecules by reducing the level of signaling factors required to activate QS processes (Beckmann et al., 2012). Thus, contrary to the prevailing opinion, there is a possibility of developing resistance to QQ therapies (Kalia et al., 2014; Liu et al., 2018; Krzyżek, 2019).

### 4.3 Microbial adhesion and evaluation of anti-adhesion potential of novel compounds

Bacteria adhere to various types of living and non-living surfaces in order to increase survival likelihood. Adhered bacteria have frequently increased access to nutrients and are more able to withstand hydrodynamic and mechanical forces of encountered physical stressors (van Loosdrecht et al., 1990; Fletcher, 1994). Moreover, bacterial adhesion is in most cases the initial step in biofilm formation (Busscher et al., 2009). For pathogenic bacterial species, adhesion is a crucial virulence factor for the human host (Busscher and van der Mei, 2012). Given that adhesion of pathogens to host cells or tissues represents the first step in bacterial infection, anti-adhesive molecules may provide a powerful prophylactic tool against infections (Wittschier et al., 2007; Krachler and Orth, 2013).

Adhesion ability determines the level of colonization of human tissue or medical devices (catheters, prosthetic implants, vascular grafts etc.) that a particular strain may achieve (Zhang et al., 2020). Bacterial adhesion is mediated by two types of interactions: non-specific physico-chemical forces, such as Van der Waals forces, electrostatic forces and hydrogen bonds, that acts initially to bring bacteria to a close contact with the adhering surface; and specific molecular interactions that enable formation of tight bonds (Boland et al., 2000; Krasowska and Sigler, 2014). Several various molecular interactions, mediated by different bacterial proteins have been described. Among the best described are *E. coli* specific structure-mediated mechanisms that often distinguish pathogenic strains from non-pathogenic microbiota-composing ones due to the ability to normally colonize *E. coli*-free compartments of the human body such as the small intestine and the urethra (Kaper et al., 2004). Those specific structures mostly form distinct morphological structures – fimbriae (pili) or fibrillae composed of specific bacterial proteins adhesins, and include several types, for example: colonization factor antigen (CFA)/III fimbriae of enterotoxigenic *E. coli* (ETEC), CFA/I fimbriae of ETEC, P pili of uropathogenic *E. coli* (UPEC), CS3 fibrillar structures of ETEC, bundle-forming pilus (BFP) of enteropathogenic *E. coli* (EPEC) which is a member of type IV pili family, curli fibres produced by a variety of pathogenic as well as non-pathogenic strains etc (Rojas-Lopez et al., 2018). Adhesion ability also presents significant virulence factor for *S. aureus* which predominantly uses MSCRAMM (Microbial Surface Component Recognizing Adhesive Matrix Molecule) family proteins for adhesion, known for the ability that a single protein can carry out multiple functions in most of the times (Foster et al., 2014). Well-known for this feature is also OmpA and its homologue proteins, which is a large protein located in outer membrane of Gram-negative bacteria, representing key virulence factor for number of pathogens such as *P. aeruginosa*, *A. baumannii* etc (Krishnan and Prasadarao, 2012; Nie et al., 2020). Ability of bacterial adhesion proteins to bind specific host cell surface proteins determines host tissue tropism of bacterial pathogens (Klemm and Schembri, 2000). Besides, bacterial pathogens can bind to extracellular matrix (ECM) proteins of the human host including collagen, elastin, fibrillin, laminin, fibronectin, vitronectin, thrombospondin, proteoglycans and hyaluronic acid (Singh et al., 2012). Being ubiquitously distributed throughout the human body, ECM which is involved in numerous functions such as structural scaffold formation, cellular signaling and solute transport, present attractive target for bacteria to adhere to a surface (Hynes, 2009). ECM adhesion is recognized as a significant virulence factor particularly in *E. faecalis* and *S. aureus* (Rhem et al., 2000; Somarajan et al., 2015). *E. faecalis* clinical isolates show different adhesive capacities toward ECM proteins, using differentially expressed adhesins such as the best described Ace adhesin – collagen I binding protein, structurally similar to collagen binding adhesin (CNA) of *S. aureus* (Rich et al., 1999; Tomita and Ike, 2004). *S. aureus* also exhibits wide range of adhesins, most of which belong to MSCRAMMs (Foster and Höök, 1998).

**Figure 2:**
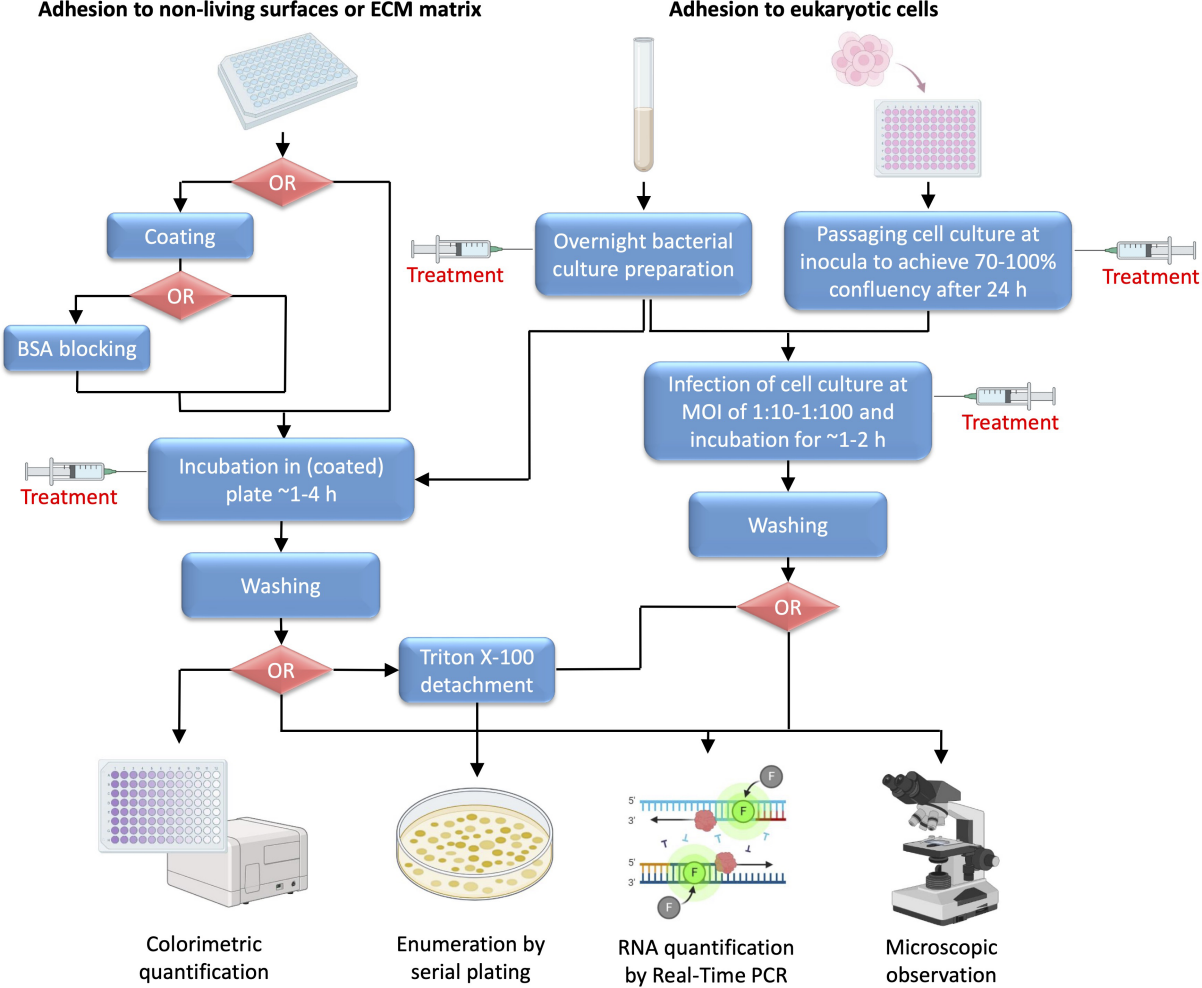
Schematic representation of adhesion testing methodology. Unless default polystyrene surface of microtiter plate is not used as a target surface, coating is the first common step for non-living surface or ECM matrix adhesion testing. Coating with non-living materials is based on the use of specific protocols for solution preparation and incubation, whereas for ECM matrix adhesion solutions of ECM matrix proteins are incubated in plate usually at 4°C overnight, with subsequent BSA blocking. Incubation of overnight bacterial culture inoculum in coated plate is carried out until the adhesion process is fully or partially complete (generally ~1-4 h), which is influenced by several factors, including specific bacterial species, surface characteristics and environmental conditions such as temperature, pH, humidity, presence of specific nutrients etc. For adhesion to eukaryotic cells, usually human cell lines are used, for example HeLa or Caco-2 cells when testing adhesion to epithelium, THP-1 or U937 cells for studying bacterial interaction with immune cells, or A549 cells suitable for respiratory bacterial adhesion studies. In addition, bacterial adhesion to fungal hyphae, such as *Candida albicans* hyphae, may be investigated. When infecting eukaryotic cells, it is important to adjust suitable MOI and generally bacterial inoculum in PBS is added at 1:10-1:100 of number of seeded human (of fungal) cells 24 h earlier. Dependent of the predicted mechanism of action, treatment with tested agent can be done in different stages, including preparation of overnight bacterial culture, preparation of cell culture monolayer, or incubation of bacterial inoculum in plate coated with desired material, protein or eukaryotic cell monolayer.

There are several ways to investigate anti-adhesive properties of novel compounds. Using various techniques, three different types of bacterial adhesion can be examined: adhesion to non-living surfaces, adhesion to eukaryotic cells (in most cases human cells), and adhesion to ECM proteins (**Figure 2**).

Adhesion to non-living surfaces is tested by simple incubation of investigated bacterial culture inoculum on selected type of surface supplemented with suitable growth medium for a short period of time (usually no longer than 2 h, before the biofilm formation starts to occur). The bottom of the microtiter plate can be coated with surface of the desired material and quantification of adhered bacteria is performed after removal of excess unbound bacterial cells (Extremina et al., 2010). The treatment agent may be added before adhesion to an overnight culture in case it is suspected that it may affect protein or RNA expression of target adhesion protein(s), or it may be incubated afterwards when adhesion ability of investigated bacteria is examined if it putatively directly interferes with the binding interaction.

When testing bacterial adhesion to eukaryotic cells, the bacterial culture and the host cell culture are prepared separately using suitable culture media. Continuous human cell lines are usually used, selected based on the known tissue tropism of investigated pathogen. First, the cell culture is prepared in plates to achieve ~70-100% confluence and then infected with a separately prepared bacterial inoculum (usually at mid-exponential phase). It is important to adjust appropriate multiplicity of infection (MOI) when adding bacterial inoculum to the cell culture monolayer. Usually, overnight culture of bacteria resuspended in Phosphate-Buffered Saline (PBS) is added at 1:10-1:100 of seeded human cells. Before adding bacterial inoculum, cell culture medium is removed from the prepared monolayers, which are then washed with PBS to remove excess of unattached cells (**Figure 2**). Since, adhesion usually occurs early, the bacteria are incubated only for 1-2 h with cell monolayer, and then washed with PBS to remove unbound bacteria (Jayashree et al., 2018; Saha et al., 2023). The tested compound is usually added to an overnight bacterial culture. It can also be added during the bacterial infection of cells if direct interference with binding interaction is suspected, but caution must be taken to ensure that the compound does not affect cell monolayer in any other way. Another possibility is to treat cell culture during the seeding if the agent potentially affects expression of eukaryotic cell molecular target of bacterial adhesion protein.

Several means of quantification of adhered bacterial cells can be utilized, based on colorimetric quantification, enumeration of viable cultivable bacterial cells following the plating of serial dilutions of bacterial cells, counting of bacterial genetic material, or microscopic observation. Simple colorimetric quantification can be used when examining adhesion to non-living materials. Caution must be taken when choosing between different staining methods, for example crystal violet or safranin stain complete biomass that is present (including live and dead cells, as well as the biofilm), whereas 3-(4,5-dimethylthiazol-2-yl)-2,5-diphenyltetrazolium bromide (MTT), 2,3-Bis-(2-methoxy-4-nitro-5-sulfophenyl)-2H-tetrazolium-5-carboxanilide (XTT), or resazurin stain only viable bacterial cells, including viable but non-culturable (VBNC) cells. The plating technique, on the other hand, enables precise counting of viable culturable cells, but excludes VBNC cells. When this technique is used to count number of bacterial cells adhered to eukaryotic cells, bacterial cell detachment using Triton X-100 must be performed prior to plating (**Figure 2**) (Extremina et al., 2010; Azeredo et al., 2017; Saha et al., 2023). More specific methods of quantification include RNA expression analysis of bacterial target DNA sequences, use of specific antibodies in an Enzyme-linked immunosorbent assay (ELISA) type of reaction, and microscopic observation or flow cytometry of differentially labeled cells (Alvarez et al., 2013; de Oliveira et al., 2016; Jayashree et al., 2018; Wang et al., 2022).

Finally, when testing adhesion to ECM proteins, microtiter plates (preferably specially designed plates with a high absorbance surface) are initially coated with investigated ECM protein. Several coating techniques have been described, among which the most frequent technique is based on the overnight incubation of the plates filled with suitable concentration of ECM protein solution at +4°C. To check whether coating was successful, Bradford, Bicinchoninic Acid Assay (BCA) or related method can be used to measure concentration of proteins in plate wells (following the washing of excess of unbound proteins) (Stoscheck, 1990). The excess of unbound proteins is washed off, and the plates are blocked using 1-2% bovine serum albumin (BSA) (**Figure 2**). The purpose of this step is to block any remaining potential bacterial binding sites, to prevent nonspecific binding when the bacterial culture is added and to ensure that all bacterial cells bind only to the tested ECM protein. The bacterial inoculum is then added and incubated under appropriate bacterial growth conditions with bound ECM proteins for a short period of time until binding is complete (~1-4 h). Finally, the excess of unbound bacteria is washed off with PBS and quantification is performed either by staining the bacteria and following colorimetric or microscopic evaluation or by serial plating following the Triton X-100 mediated detachment (Smani et al., 2012; de Oliveira et al., 2016; Ušjak et al., 2021).

Number of papers have demonstrated significant inhibitory activities against microbial adhesion for various novel compounds. Of note, *A. baumannii* OmpA inhibiting synthetic cyclic hexapeptide was found to display powerful adhesion limiting abilities against several Gram-negative bacteria. To mimic the interaction between bacteria and the respiratory epithelium, the authors used human lung epithelial cells (A549), which they treated with a non-cytotoxic concentration of the selected compound, and a fibronectin-binding assay (Vila-Farrés et al., 2017). Similarly, Lee et al. (2009) specifically used human gastric epithelial cells (AGS), human oral epidermoid carcinoma cells (KB), and mouse embryonic fibroblast cells (NIH3T3), to demonstrate potent anti-adhesive effect of a green tea extract against *H. pylori*, *Porphyromonas gingivalis*, and *Cutibacterium acnes* or *S. aureus*, respectively. Importantly, in addition they confirmed that the tested extract did not induce hemolysis or affect cell viability. In another interesting study, anti-adhesive properties of chrysophanol-decorated silver nanoparticles (CP-AgNPs) coating of polystyrene and silicone surfaces were demonstrated against *P. aeruginosa* and *E. coli*. This study utilized light microscopy, crystal violet assays, and SYTO-9 staining for fluorescence microscopy to quantify and visualize bacterial adhesion (Prateeksha et al., 2021). Although microscopy represents powerful tool for morphological observation of the adhesion process, it does not provide quantitative, single-cell and real-time high-throughput data. Therefore, Scharenberg et al. (2011) designed a flow cytometry-based assay using the human urinary bladder cell line 5637 to demonstrate the potential of various mannose derivatives and analogs as FimH antagonists in preventing UPEC adhesion. The assay involved infecting 5637 cells with UPEC, testing various FimH antagonists, and adherence was measured by flow cytometry. In addition, the authors conducted an invasion assay to further assess bacterial internalization.

## 5 Evaluating safety of novel compounds

### 5.1 Cytotoxicity of novel compounds

The toxicity of novel compounds is routinely assessed using cytotoxicity assays on normal cell lines. Normal fibroblasts are usually selected since they are easy-to-grow in culture and are highly sensitive to cytotoxic activities. Most commonly, L-929 mouse lung fibroblasts are used, as they share many of the common basic mechanisms with both specialized and non-specialized human cell types (Wieslander et al., 1991; Dang et al., 2017). Also, BALB/3T3 clone A31 fibroblasts originating from mouse embryos, MRC-5 and WI-38 human lung fibroblast cell lines, Vero and BHK-21 cell lines derived from the kidney tissue of adult African green monkeys and Syrian golden hamsters, respectively, and V-79 379A hamster lung fibroblasts are also recommended by ISO experts as suitable for cytotoxicity testing (ISO 10993-5: 2009). However, other cell lines such as corneal epithelial cells, Chinese hamster ovary cells, canine renal cells and HeLa cells have also been used (Ballantyne, 2006). Preferably, novel compounds should be tested on several cell lines with different characteristics.

The most used test is the MTT assay (Ušjak et al., 2023). The MTT assay is based on the formation of insoluble violet colored formazan crystals from the yellow MTT tetrazolium salt reagent, likely by the activity of NADH- and NADPH-dependent mitochondrial oxidoreductases of the live cells (Berridge and Tan, 1993). In general, appropriate cell number is seeded so to achieve 60-80% confluence after 24 h of incubation. Cell overcrowding may lead to a loss of linearity and imprecise readings due to the contact-dependent growth inhibition and slowed down metabolism in a portion of cell population (van de Loosdrecht et al., 1991). The cell monolayers are then treated with the tested agent for a specific period of time (usually 24-48 h), after which the MTT reagent is added to the reaction at a final concentration of 0.2-0.5 mg/mL. In this step, caution must be taken and considered whether the MTT reagent interferes with the tested agent, for example if it is a chemical that can reduce tetrazolium salts as well, in a non-enzymatic manner (Peng et al., 2005). If so, there are several possibilities to avoid or minimize this issue, such as: medium can be discarded and treated cell monolayer supplemented with fresh media with added MTT reagent; or appropriate controls without cell monolayers can be used. Replacing the medium is also a method of choice if incubation with the treatment agent is prolonged (for example 48 h) as the pH may change or essential nutrients of the medium may be depleted, which may affect the ability of the cells to reduce MTT and lead to inaccurate readings. Incubation with the MTT reagent is performed in the dark for 1-4 h, by periodic monitoring of formation of formazan crystal deposits. Insoluble formazan products accumulate inside cells, as well as extracellularly in the culture medium. In case of observation of large extracellular crystal deposits incubation should be ceased, since further incubation may likely increase loss of linearity, due to the cytotoxic nature of crystal deposits and accumulation of the formazan crystals formed by spontaneous reduction of tetrazolium salts (Lü et al., 2012). Finally, formazan products are solubilized by using dimethyl sulfoxide or other appropriate solvent/detergent (acidified isopropanol, dimethylformamide, sodium dodecyl sulphate, etc.) and the absorbance is measured at 570 nm, with the background measurement at 630 nm.

Besides MTT, other tetrazolium salts (e.g. XTT, 3-(4,5-Dimethylthiazol-2-yl)-5-(3-carboxymethoxyphenyl)-2-(4-sulfophenyl)-2H-tetrazolium (MTS), and water-soluble tetrazolium-1 (WST-1)) and resazurin can also be used to measure cytotoxic activity (Malich et al., 1997; Ngamwongsatit et al., 2008; Bila et al., 2021). These compounds do not require addition of organic solvent or detergent for solubilization; therefore, cells do not have to be lyzed for the purpose of absorbance measurement and this allows kinetic monitoring of the tested agent effect at the different timepoints. MTS, XTT and WST-1 tetrazolium salts need to be used in combination with intermediate electron acceptors (e.g. phenazine methyl sulphate (PMS) and phenazine ethyl sulphate (PES)), whereas resazurin solution can be directly added into cells in culture. The advantage of the resazurin reduction assay is also that the formation of the pink-colored reduction product (resorufin) can be measured both colorimetrically and by recording the change in the fluorescence signal at 560 nm excitation and 590 nm emission wavelengths. Measuring the fluorescence signal usually offers higher sensitivity, unless tested compounds exhibit similar fluorescent emissions resulting in the interference. Besides, the resazurin reduction assay is fast, relatively inexpensive, and more sensitive than tetrazolium assays. However, caution must be taken on direct cytotoxic effects of resazurin (Costa et al., 2021).

Further, cytotoxic activity can be assessed by measuring protease activity or the ability to synthesize adenosine triphosphate (ATP) in cell culture. The protease assay is based on the generation of fluorescent molecule from glycyl-phenylalanyl-aminofluorocoumarin (GF-AFC) substrate by the activity of cytoplasmic aminopeptidase. Advantages of this assay include the shorter incubation time (0.5-1 h) and the low toxicity of the GF-AFC substrate, which enables use of subsequent assays on treated cell culture, e.g. to examine mechanisms leading to cell death (Niles et al., 2009; Armento et al., 2020). The ATP assay utilizes luciferase enzymes and ATP as a cofactor to create luminescent signal from luciferin substrate. Being the fastest and the most sensitive, this type of assay, along with its modifications, provide an option for high-throughput screening (Crouch et al., 1993; Gavanji et al., 2023). Finally, another high-throughput cytotoxicity test is based on flow cytometry of differentially labelled cells. The most used staining method is based on the use of inexpensive propidium iodide which binds to the DNA in cell nuclei of the damaged/dead cells with permeable plasma membranes and is usually coupled with annexin V staining of the damaged plasma membranes. Another advantage of this test is that it offers differentiation between apoptotic and necrotic cells, thus yielding information on cell death mechanism (Edwards et al., 2007; Rieger et al., 2011; Crowley et al., 2016).

Cytotoxicity assays have been widely used by researchers investigating the antimicrobial properties of novel compounds to gain valuable initial insights into potential adverse effects and safety profiles of the compounds. One group of authors synthesized series of chalcone derivatives with potent antimicrobial activities, for which they subsequently evaluated both cytotoxicity and anticancer activity against A549 lung adenocarcinoma, HepG2 hepatocellular carcinoma, and C6 glioma cell lines, using the MTT assay. The authors further tested compounds against NIH/3T3 healthy cells to demonstrate preference for inducing cytotoxic effects in cancer cells over normal, healthy cells. It is noteworthy that these authors also tested the genotoxicity of the two most promising compounds using the Ames assay, which is described in the next section (Özdemir et al., 2017). On the other hand, the authors, who investigated the antimicrobial activity of β-hairpin antimicrobial peptides (AMPs), decided to use resazurin (Alamar Blue Assay) to determine cytotoxicity in a non-destructive measurement with increased sensitivity and linearity as well as ease of use. They evaluated therapeutic potential based on the therapeutic index, calculated as the ratio of average toxicity against HEK293 and HepG2 human cell lines to median MIC against bacteria and fungi. The findings highlighted the delicate balance between antimicrobial efficacy and cytotoxicity, essential for the development of selective AMPs (Edwards et al., 2016). Another example is an investigation of cytotoxicity of the ordered mesoporous silica materials like MCM-41 and SBA-15, intended for potential incorporation into oral drug formulations, considering their feasibility in drug delivery applications. In this study, authors used multiple assays on human colorectal adenocarcinoma cells (Caco-2) to obtain comprehensive cytotoxicity profiles. They used fluorescent cell viability assay (AFC) utilizing the CellTiter-Fluor™ substrate, to provide a fluorescence signal proportional to live-cell protease activity, indicating cell viability. The luminescent cell viability assay (ATP) quantified viable cells based on ATP production, offering insights into metabolic activity. Flow cytometry analyzed cell viability with propidium iodide staining. The Caspase-3/7 activity assay measured apoptosis as a marker of programmed cell death. Reactive oxygen species (ROS) were detected by DCF-DA and DHE staining (fluorescent probes), which indicate oxidative stress. Finally, the GF-AFC protease assay specifically addressed cell membrane integrity, revealing the cytotoxic effects of mesoporous silica particles on Caco-2 cells over varying concentrations and incubation times (Heikkilä et al., 2010).

However, although *in vitro* cytotoxicity assays provide valuable insights into the potential toxic effects of substances, offering a controlled environment for initial safety assessments, their accuracy in predicting real-world outcomes may be limited due to the simplified nature of *in vitro* models, which lack the complexity of intricate physiological and metabolic interactions in the human body. Factors such as tissue-specific responses, systemic effects, and the influence of the immune system are challenging to replicate accurately *in vitro*, emphasizing the importance of complementing *in vitro* data with *in vivo* studies for a more comprehensive understanding of cytotoxicity and its real-world implications (Kroll et al., 2009; De Jong et al., 2020).

### 5.2 Evaluation of mutagenic potential of novel compounds using Ames test

**Figure 3:**
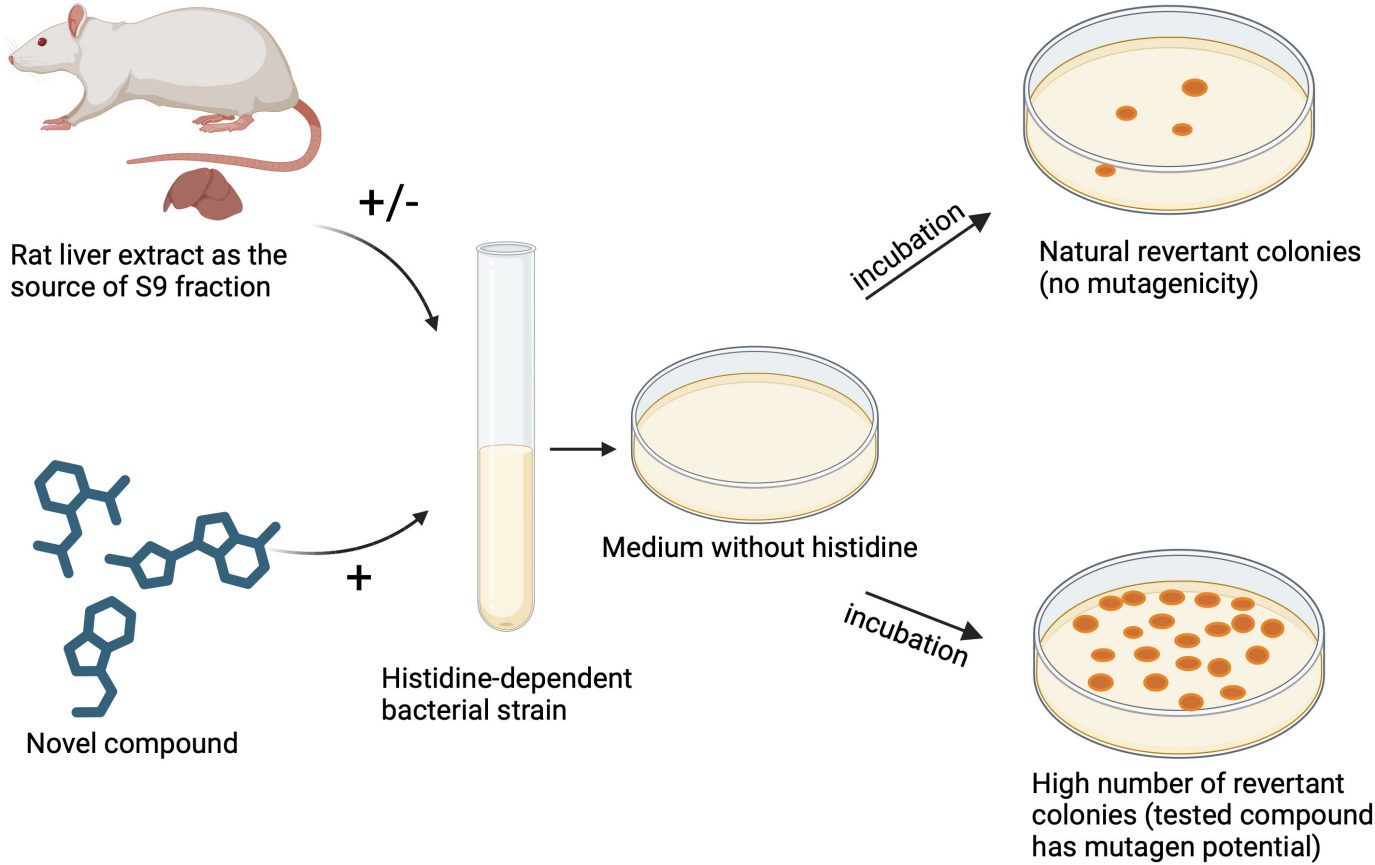
Evaluation of mutagenic potential of novel compounds using Ames test. Figure made by BioRender.com.

If a new active substance has an antimicrobial and/or antivirulence effect, it should first be tested to ensure that it has no mutagenic potential that could affect human cells before it can be used as a novel drug. The biological assay widely used to predict the mutagenic potential of newly synthesized compounds is the Ames test. This assay involves bacteria of different strains of *Salmonella typhimurium* and *E. coli* (Vijay et al., 2018). The Ames test is highly used in pharmaceutical industry as almost all new pharmaceutical chemicals and substances are tested by this assay, and the data obtained from the test are used for submission to regulatory agencies for registrations of new drugs. The great advantage of the Ames test is that it is faster and cheaper than animal testing. The assay was named after its inventor Bruce Nathan Ames who established the protocol in 1975, which was reappraised by him and his followers in 1980s (Maron et al., 1981). Bruce Ames developed a simple, rapid and robust test procedure and found that most carcinogens known at the time were mutagenic according to this test (Smith et al., 2021). The Ames test uses a number of *Salmonella* strains that carry mutations in various genes in the histidine operon that are involved in histidine synthesis meaning that these strains are unable to synthesize histidine (histidine-dependent), and therefore unable to grow and form colonies on agar media without histidine (**Figure 3**) (Mortelmans and Zeiger, 2000). In the presence of the mutagenic chemicals, these mutant, histidine-dependent bacterial strains revert to their original state and restore their ability to produce histidine, which is why the test is often referred to as a “reversion test” (**Figure 3**) (Azahar et al., 2019). As a positive result of the test, the growth of bacterial colonies is observed on agar plates with low concentration of histidine. Normally, the tested compound and the selected bacterial strains are cultured for 48 h before the bacterial colonies are calculated on Petri dishes (Goyal et al., 2022).

Recommendation of the test is that at least five strains of bacteria should be used, including four different strains of *Salmonella typhimurium* (TA1535; TA1537 or TA97a or TA97; TA98; and TA100) as for these strains have been shown to have several features that make them more sensitive to detect mutagens (OECD, 2020). However, it is known that these *S. typhimurium* strains may not detect certain oxidizing mutagens which can be detected by *E. coli* WP2 strains carrying mutations in various genes of the tryptophan operon or *S. typhimurium* TA102 (Wilcox et al., 1990).

Therefore, the recommended combination of strains is:

1. *S. typhimurium* TA1535, and
2. *S. typhimurium* TA1537 or TA97 or TA97a, and
3. *S. typhimurium* TA98, and
4. *S. typhimurium* TA100, and
5. *E. coli* WP2 uvrA, or *E. coli* WP2 uvrA (pKM101), or *S. typhimurium* TA102 (OECD, 2020).

Some examples of compounds that have been evaluated using the Ames test are: nitrofuranylamides as anti-tuberculosis agents (Hurdle et al., 2008), non-basic melanin-concentrating hormone receptor 1 (MCHR1) antagonists such as 1-(2-cyclopropyl-3-methyl-2H-indazol-5-yl)-4-{[5-(trifluoromethyl)thiophen-3-yl]methoxy}pyridin-2(1H)-one (Igawa et al., 2016), derivatives of 5-fluorouracil which is one of the first line drugs for the systemic therapy of solid tumors such as breast, colorectal, esophageal, gastric, pancreatic, head and neck tumors, such as 5-fluorouracil derivative 1-[{1’-(2″,3″,4″,6″-tetra-O-acetyl-β-d-glycopyronosyl)-1’H-1’,2’,3’-triazole-4’-yl} methyl]5-fluorouracil (Köksal Karayildirim et al., 2018), novel multipotent compounds modulating endocannabinoid and dopaminergic systems (Grillo et al., 2019), novel selenocompounds (Marć et al., 2022), alkyl-nitrosamines- N-nitrosodimethylamine (NDMA) and N-nitrosodiethylamine (NDEA), and N- nitrosamines (Trejo-Martin et al., 2022; Thomas et al., 2023) etc.

The disadvantage of this test may be the fact that the assay is performed using prokaryotic instead of eukaryotic cells. Ames assay consists of *Salmonella typhimurium* strains and so it is not a perfect model for human cells (Vijay et al., 2018). Some chemicals display the mutagenicity only after being metabolized by enzymes that belong to the cytochrome P450 family and these enzymes are not present in bacterial cells (Azahar et al., 2019). To overcome this issue, the assay is performed with S9-based metabolic activation system (**Figure 3**). Mice liver S9 hepatic fraction is used to minimize the mammalian metabolic activations formed in the hepatic system so that the mutagenicity of metabolites can be assessed. The S9 fraction is supernatant fraction obtained from the rat liver homogenate after centrifugation at 9000 g (Vijay et al., 2018). There are several differences between human and mice metabolism which can affect the mutagenicity of testing substances, and the newly synthesized compound should be tested for mutagenic potential with bacteria in the presence and the absence of S9 fraction (**Figure 3**) (Vijay et al., 2018; OECD, 2020). At least five different concentrations of the test substance should be used and the recommended maximum concentration for soluble non-cytotoxic substance is 5 mg/plate or 5 μl/plate (OECD, 2020).

The data of the test should be presented as the number of reverted colonies per plate compared to the negative control where spontaneously reverted colonies may occur. If needed, statistical methods can be used to interpret the results, although most tests give clearly positive or negative results (Vijay et al., 2018; OECD, 2020).

The Ames test is one of the most used tests in toxicology and almost all new pharmaceutical substances and chemicals used in industry are tested with this assay using specific microorganisms. The absence of mutagenic potential of novel compound tested with the Ames assay is an indicator that the compound is not mutagenic to human cells.

## 6 Conclusion

**Figure 4:**
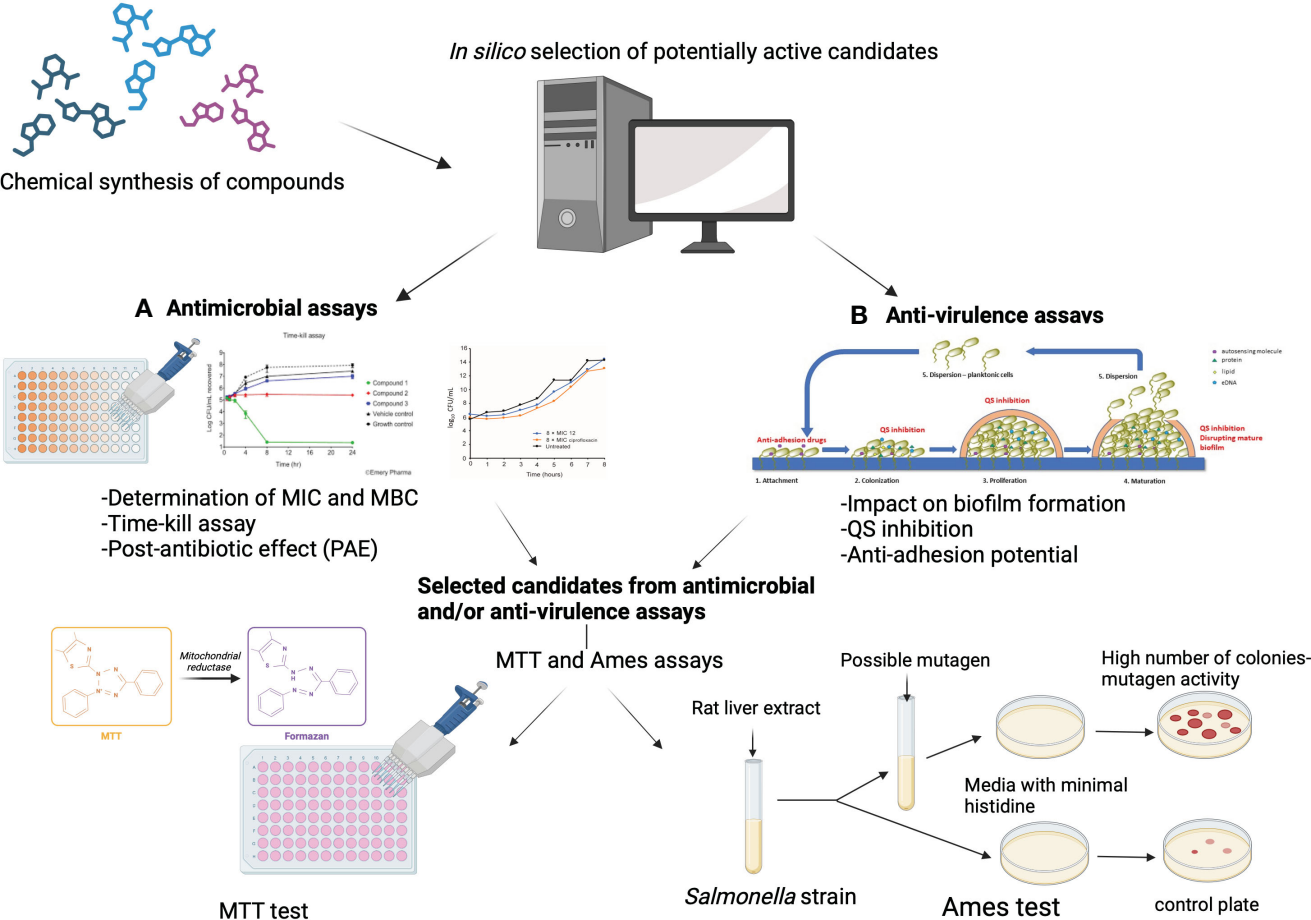
The key steps in the evaluation of newly synthesized compounds for their antimicrobial and/or anti-virulence activity using *in silico* and *in vitro* methods and toxicity testing to select potential drug candidates. Abbreviations: MIC - Minimal Inhibitory Concentration; MBC - Minimal Bactericidal Concentration; QS - Quorum sensing; MTT - 3-(4,5-dimethylthiazol-2-yl)-2,5-diphenyltetrazolium bromide. Figure made by BioRender.com.

The emergence and spread of antibiotic-resistant bacteria is a global threat and novel compounds with anti-bacterial and/or anti-virulence activity are urgently needed. It is now clear that there are two possible approaches to kill or attenuate resistant bacterial cells. The traditional approach is to target bacterial vital components or processes similarly to existing antibiotics. The alternative, and perhaps the more intelligent approach is to disarm resistant bacterial cells and suppress their virulence factors involved in disease development. In this review *in silico* and *in vitro* screening strategies of newly synthesized compounds for anti-bacterial and anti-virulence activities are presented. To the best of our knowledge, the most important aspects of screening newly synthesized compounds are summarized for the first time in this paper: a) *in silico* methods (QSAR and molecular docking); b) antimicrobial assays (determination of MIC, MBC and time-kill assay); c) tests that can help to determine the microbial potential to develop resistance to novel compounds (post antibiotic effect and development of spontaneous mutations using serial passages); d) anti-virulence assays (impact on biofilm formation, QS and adhesion to different substrates); and e) important toxicity assays for the potent new compounds (cytotoxicity assay and Ames test) (**Figure 4**). This review of methods may provide useful guidelines for all researchers working in the field of discovering new antimicrobial agents as well as for microbiologists to use appropriate assays to evaluate new compounds. An important fact is that a new compound may exhibit antimicrobial activity (e.g. it is possible to determine the MIC value) and anti-virulence activity. On the other hand, compounds that do not target structures inside of the bacterial cells (MIC value cannot be determined), may exhibit potent anti-virulence activity, for example by targeting biofilm components or by affecting human biomolecules crucial in adhesion process. Therefore, it is of great importance to use both, anti-bacterial and anti-virulence approaches, when examine novel compounds as potential new therapeutics. Successful application of the methodological pipeline described above could lead to an appropriate selection of drug candidates for further preclinical and clinical trials.

## Author contributions

BF: Conceptualization, Formal Analysis, Methodology, Resources, Software, Visualization, Writing – original draft, Writing – review & editing. DU: Conceptualization, Methodology, Visualization, Writing – original draft, Writing – review & editing. MR: Conceptualization, Methodology, Visualization, Writing – original draft, Writing – review & editing. SO: Conceptualization, Methodology, Writing – original draft, Writing – review & editing. MM: Conceptualization, Funding acquisition, Methodology, Supervision, Writing – original draft, Writing – review & editing.
